# Improving the production of podophyllotoxin in hairy roots of *Hyptis suaveolens* induced from regenerated plantlets

**DOI:** 10.1371/journal.pone.0222464

**Published:** 2019-09-12

**Authors:** Crescencio Bazaldúa, Alexandre Cardoso-Taketa, Gabriela Trejo-Tapia, Brenda Camacho-Diaz, Jesús Arellano, Elsa Ventura-Zapata, María Luisa Villarreal

**Affiliations:** 1 Departamento de Biotecnología, Centro de Desarrollo de Productos Bióticos, Instituto Politécnico Nacional, Yautepec, Morelos, México; 2 Laboratorio de Plantas Medicinales, Centro de Investigación en Biotecnología, Universidad Autónoma del Estado de Morelos, Cuernavaca, México; 3 Laboratorio de Botánica Estructural, Centro de Investigación en Biotecnología, Universidad Autónoma del Estado de Morelos, Cuernavaca, México; National University of Kaohsiung, TAIWAN

## Abstract

Ten *Hyptis suaveolens* hairy root lines were established by infecting nodal explants with K599+pGus-GFP+ and ATCC15834+pTDT strains from *Agrobacterium rhizogenes*. Genetic transformation was confirmed by epifluorescence and plagiotropic hairy root growth in absence of growth regulators. Cytotoxicity was determined using the sulforhodamine B method, and the production of podophyllotoxin (PTOX) was measured by high performance thin layer chromatography scanning. Through these methodologies, HsTD10 was identified as the hairy root line with the highest cytotoxicity and PTOX production, which was corroborated by liquid chromatography-mass spectrometry and micrOTOF-Q II. A suspension culture of HsTD10 was established in which PTOX and carbohydrate consumption during growth kinetics were quantified by high-performance liquid chromatography. Procedures to increase the production and retrieval of PTOX in the HsTD10 line included selection of culture medium, addition of thiamine, and modification of the PTOX extraction method. The best combination of these variables was MS medium at 75% of its components with the addition of 2 mg L^-1^ of thiamine, extraction with methanol-dichloromethane, and sonication at 40 ± 5°C. During kinetics, growth-associated PTOX accumulation was observed. The specific growth rate (μ) was 0.11 d^-1^. The highest concentration of PTOX obtained with HsTD10 (5.6 mg g^-1^ DW) was 100 times higher than that reported for roots of wild plants and 56 times higher than that for *in vitro* nontransformed roots of *H*. *suaveolens*.

## Introduction

Podophyllotoxin (PTOX) is a natural 2,7'-cyclolignan used to obtain the semisynthetic derivatives Etoposide®, Teniposide® and Etopophos®, which are medicines widely used in cancer chemotherapy. The main source of PTOX is the species *Podophyllum hexandrum* [1], a wild plant native to the Himalayas, which has been overexploited and is currently endangered [2]. Annual PTOX production is 50–80 tons per year; however, demand exceeds 100 tons per year [3].

Chemical synthesis of PTOX is highly difficult and expensive due to the complexity of its molecular structure. The search for new natural sources that produce PTOX is mandatory, as is research for biotechnological alternatives, to achieve stable and controllable production of this compound [4]. Today, the most important PTOX producer species are *Podophyllum hexandrum*, *Juniperus bermudiana*, and *Podophyllum peltatum*, which produce 43, 22.6, and 4.7 mg g^-1^ dry weight (DW) of PTOX, respectively [1, 5, 6].

The highest concentration of PTOX obtained from *in vitro* plant cultures (8.0 mg g^-1^ DW) was reported in cell suspension cultures from the selected line Herm A of *Linum album* [7], whereas the second and third were obtained from cell suspension cultures of *Podophyllum hexandrum* and *P*. *peltatum* of 7.9 and 5.9 mg g^-1^ DW, respectively [8, 9]. A high level of production of PTOX has been reported in hairy root cultures of *Linum album* and *L*. *flavum* at concentrations of 3.5 to 15 mg g^-1^ DW [10–13].

*Hyptis suaveolens* is a wild shrub native to Mexico that was employed throughout history, mainly for the treatment of gastric and bile problems, cancer, fever, spasms, cough, malaria, yellow fever and other diseases [14]. As we previously reported, this plant produces PTOX [15], and its roots growing *in vitro* accumulate more PTOX than wild roots (0.57 vs 0.05 mg g^-1^ DW, respectively) [16]. We also ascertained that the hairy roots of this plant, growing in suspension cultures in Gamborg’s B5 culture medium without hormones, produce PTOX or its analogs [17].

The aim of this research was to establish and select hairy root culture lines of *Hyptis suaveolens*, producing PTOX, as well as optimizing culture conditions to increase PTOX accumulation. This research demonstrates that the most effective factors for determining a good yield of PTOX were the culture medium MS at 75% of its components, amended with 2 mg L^-1^ of thiamine. Similarly, sonication at 40 ± 5°C using methanol-dichloromethane for extraction increased PTOX yield. The accumulation of PTOX in the hairy root suspension cultures of *H*. *suaveolens* was growth-associated.

## Materials and methods

### Plant material

Plants and seeds of *Hyptis suaveolens* were collected in Merida, Yucatan, Mexico, in 2006 and authenticated by German Carnevalli, Scientific Research Center of Yucatan Herbarium. A specimen of the plant was deposited (voucher number CICY 7086). The seeds were carefully washed under running tap water for 10 min and surface-sterilized by immersing in ethanol at 70% (v/v) for 1 min and in sodium hypochlorite at 1.5% (v/v) for 5 min. The sterilized seeds were immediately rinsed three times with sterile distilled water for 5 min. Subsequently, the seeds were placed in glass vessels containing Murashige and Skoog medium (MS, Sigma) [18], amended with sucrose 3% (w/v), and 2.5 g L^-1^ water-phytagel for germination. The plantlets were maintained until they were 5 cm in length in a growth chamber at 24 ± 2°C with a photoperiod of 16/8 h light/darkness. Light sources were cool white lamps of 40 watts.

### Obtaining and multiplying regenerated plantlets

The *in vitro* multiplication of plantlets was achieved by placing nodal segment explants measuring 3 cm in length under the same conditions described previously. During plantlet regeneration, three concentrations were evaluated: three-quarters strength (TQS), half strength (HS), and full strength (FS) of MS medium. Plantlet height and root length were registered.

### Induction and selection of hairy roots

Root induction was performed by infection of nodal explants with two strains of *Agrobacterium rhizogenes*, K599+pGus-GFP+ (facilitated by Dr. Federico Sanchez†, IBT-UNAM), and ATCC15834+pTDT, obtained in a previous work [17]. One hundred forty-four putative hairy roots were placed in Petri dishes with TQS MSB5 medium (M0404-Sigma), which contains the same amount of inorganic salts and myo-inositol as MS medium, but it is supplemented with vitamins, as described by Gamborg (B5 medium): nicotinic acid (1 mg L^-1^), piridoxine (1 mg L^-1^), and thiamine (10 mg L^-1^). This medium was supplemented with sucrose (3%, w/v) and phytagel (2.5 g L^-1^) without hormones. Putative hairy roots were maintained under these conditions for 45 days.

From these cultures, ten hairy root lines were selected based on their fast autonomous growth, hairy high branched appearance and plagiotropic growth to corroborate their genetic transformation, cytotoxic activity, and PTOX production. These selected hairy root lines (HR-lines) were transferred to TQS MSB5 liquid culture medium and kept at constant agitation of 110 rpm under the same light, temperature and humidity conditions described for plantlet growth.

#### Genetic transformation confirmation

To observe the fluorescence, 1 cm of the roots was cut and directly placed on microscope slides. An epifluorescence microscope with filters for blue and green light (460–490 nm, and 500–550 nm) (Nikon, Eclipse 80i, Tokyo, Japan) with an adapted camera (3CCD MTI, DC330, Michigan, USA) made it possible to corroborate hairy root transformation by *Agrobacterium* strains expressing the fluorescent green protein (GFP) or the tomato deaminase threonine protein (TDT- red fluorescence).

### PTOX extraction

Biomasses of ten initially tested HR lines were filter-washed, oven dried at 60°C for 72 h, finely ground, sieved through a 53-micron mesh, and extracted. Three methods of extraction were tested: 1) methanol-dichloromethane (MD-method) [19]: briefly, 2 mL of 80% (v/v) methanol was added to 100 mg of biomass dry weight (DW) and sonicated for 1 h, 4 mL of dichloromethane and 4 mL of water were added and vortexed, and the organic phase was finally recovered and dried; 2) chloroformic method: 10 mL of 100% chloroform was added to 100 mg of biomass DW, stirred for 1 h, and dried; and 3) modified MD method, where the modification of the MD method [19] involved sonication at 40 ± 5°C. All extracts were solubilized in HPLC grade MeOH and syringe-filtered (0.45 μm, Millex-Hv, Millipore) for PTOX identification through HPTLC, HPLC or micrOTOF QII.

### Identification of PTOX utilizing HPTLC-Scan

Initially, PTOX was identified using the corresponding standard (Sigma) through high performance thin layer chromatography (HPTLC-Scanner TLC-3 CAMAG), as described in 2017 by Kamal et al. [20]. The retention factor from standard (PTOX Sigma) spot was compared with those spots from the extracts obtained through the MD and chloroformic methods. HPTLC scanning of these spots complemented the results.

### Cytotoxic evaluation

To evaluate cytotoxicity, two types of extracts obtained through the MD and chloroformic methods from ten HR lines were tested. The cytotoxicity of crude extracts was determined using the sulforhodamine B method (SRB) [21] by quantifying the half-maximal inhibitory concentration (IC_50_) on four human carcinoma cell lines obtained from ATCC (HF6 = Colon, MCF7 = Breast, PC3 = Prostate, and SiHa = Uterus). To continue exploring alternatives for increasing PTOX production, the most cytotoxic HsTD10 HR-line was selected, as established by the US NCI, a plant-screening program for crude extracts (IC_50_ values less than 20 μg mL^-1^) [22]. The positive controls were standards of etoposide and PTOX (Sigma).

### Identification of PTOX using HPLC-MS and MicrOTOF-Q II in the HsTD10 HR-line

#### HPLC-MS

To quantify PTOX and to include information obtained from previous experiments, extracts were obtained through the chloroformic and modified MD methods.

The presence of PTOX in the extracts was verified by HPLC-MS (negative mode). The retention time of the peak, UV absorption spectrum and m/z 413 molecular ion of the standard PTOX (Sigma) were compared with those recorded for extracts from the HsTD10 selected HR-line. The HPLC-MS system (Shimadzu, Tokyo, Japan) was equipped with a system controller CBM-20^a^, 2 binary pumps LC-20AD, degasser DGU-20A3, autosampler SIL-20AC, column oven CTO20A, UV-Vis diodes detector SPD-M20A, mass spectrometer (LCMS-2020), ionization source with electro spray (ESI), software version 5a. Dry gas (N2): 10 L min^-1^ was employed. Also, scan m/z 330–440 nm, nebulizer gas flow 1.54 L min^-1^ was employed. A Zorbax- Eclipse Plus 4.8 x 150 mm, 5 μm, RP C-18 column was connected to a guard column. The injection volume was 10 μL, and a flow of 0.8 mL min^-1^ at 25°C was employed. An isocratic elution of the mobile phase consisting of methanol-acetonitrile-water-acetic acid (20:30:50:0.1) was used.

#### MicrOTOF-Q II and competitive fragmentation modeling for metabolite identification platform (CFM-ID)

The identification of PTOX in the HsTD10 HR-line extract was corroborated by micrOTOF- Q II 10392 by obtaining the ion m/z 415 (positive mode) of the standard PTOX (Sigma) and of the extract. The MS/MS partitioning profile of the m/z 415 and m/z 437 corresponded to PTOX-Na adduct (Na monoisotopic weight 23). The system was equipped with a micrOTOF-Q II 10392 (Bruker Daltonik, Germany), electrospray ionization source, set nebulizer: 0.4 bar, and drying oven at 180°C. Analysis was performed at 50–3000 m/z, capillary set 4500 V—500 V, and set of dry gas 4.0 L min^-1^. The predictive structures of the MS/MS partitioning profile were established utilizing the CFM-ID platform from Wishart-lab (http://cfmid3.wishartlab.com), which is referred to in the pubchem-ncbi site [23].

### Modifying culture conditions for PTOX accumulation in HsTD10 HR-line

#### Culture medium and method of extraction

The selected HsTD10 HR-line was cultured on three different media (MS, B5, and MSB5) at TQS for 30 days to evaluate root growth and PTOX production. Based on the results, the medium selected to increase PTOX production was MS at TQS, and the extraction was performed using the modified MD method (sonication at 40 ± 5°C).

#### Effect of thiamine addition

The effect of six different doses of thiamine (0.5, 1, 2, 4, 8, and 10 mg L^-1^) added to TQS MS medium was evaluated.

### Kinetic studies

Growth and metabolite production was evaluated using Erlenmeyer flasks of 125 mL containing 30 mL of culture medium (MS amended with 2 mg L^-1^ of thiamine and supplemented with sucrose at 3%, w/v), where 1.3 g L^-1^ (DW) of the HR-line HsTD10 were inoculated and then placed under constant agitation at 110 rpm. The following variables were measured every four days (three replicates) for 48 days: dry weight (fresh weight dried at 60°C for 72 h), pH, conductivity (portable conductivity meter HM digital model AP2 precision ± 2%, resolution 1 μS), medium carbohydrates and PTOX production.

Quantification of medium carbohydrates and PTOX was performed by HPLC. Duplication time was obtained by applying the formula TD = LN (2)/ μ, where “μ” is the slope grade from LN (final dry weight (DWf)–initial dry weight (DWi)/ final time (Tf)–initial time (Ti)). The specific rate growth (μ) is defined by μ = LN(2)/TD.

#### Quantification of PTOX

This analysis was performed by the calibration of a Waters controller 600 HPLC-DAD analysis (Waters. Millipore Corp., Waters Chromatography Division, Milford, Ma, USA). The HPLC system was equipped with a Waters 600E multisolvent delivery system with a Waters W996 diode array detector, autosampler (Waters 717 Plus), and Software Millenium 32 (Waters). The analytical HPLC separations were carried out on a Zorbax eclipse RP C18 (150 mm x 46 mm x 5 μm) column. The mobile phase consisted of an isocratic elution with methanol-acetonitrile-water (20:30:50) and a flow rate of 0.8 mL min^-1^. The standard PTOX was acquired from Sigma.

#### Carbohydrate quantification

To evaluate the consumption of carbohydrates during kinetics, three carbohydrate standards (sucrose, fructose, and glucose from Sigma) were utilized. Two milliliters of the culture medium were collected every four days for 48 days and frozen until use. Samples of culture medium were diluted at 6% (v/v) with HPLC grade water and then syringe-filtered (0.45 μm, Millex-Hv, Millipore). Subsequently, 10 μL of the dilution was injected directly into the HPLC column. The calibration curve and retention time of the peak from each standard were established by HPLC (Waters, Millipore Corp., Waters Chromatography Division, Milford, Ma, USA). The HPLC system was equipped with a Waters 600E multisolvent delivery system and a refractive index detector (Waters 2414). The analytical HPLC separations were performed using a CAPCELL PAK UG NH2 (150 mm x 46 mm x 5 μm) column (Shiseido, Japan). The mobile phase consisted of an isocratic elution with acetonitrile-water (75:25) at a flow rate of 0.7 mL min^-1^.

### Statistical analysis

Results analyses were performed using ANOVA and posttest HSD of Tukey with a p < 0.01 by the VassarStats- online, Website for Statistical Computation.

## Results

### *In vitro* regeneration and effect of medium concentration on *Hyptis suaveolens* plantlets

The concentration of MS [18] culture medium significantly influenced the budding and length of the regenerated plantlet roots, which grew faster on TQS MS (MS 5519 Sigma). Regarding the height of plantlets regenerated in full strength MS and those regenerated on TQS MS medium, significant differences (p<0.01) were observed after 20 days. Nevertheless, root sprouting and root length were significantly different (p<0.01) from day 10 onwards (Table 1).

**Table 1:** Effect of MS culture medium concentration on the growth of *Hyptis suaveolens* plantlets.

	Plantlets height (cm)			Root length (cm)		
	MS medium concentration					
Days	HS^1^	TQS^2^	FS^3^	HS^1^	TQS^2^	FS^3^
10	0.18 ± 0.08 a	0.82 ± 0.15 a	0.75 ± 0.14 a	0	0.42 ± 0.1 a	0
15	0.48 ± 0.09 a	1.69 ± 0.25 a	1.59 ± 0.22 a	0	1.09 ± 0.19 a	0
20	0.87 ± 0.13 c	3.15 ± 0.24 a	2.66 ± 0.27 b	0	2.08 ± 0.23 a	0
25	1.22 ± 0.15 c	4.84 ± 0.18 a	4.07 ± 0.31 b	0	3.01 ± 0.24 a	0.56 ± 0.08 b
30	2.06 ± 0.19 c	6.63 ± 0.33 a	5.86 ± 0.37 b	0.37 ± 0.008 c	5.95 ± 0.4 a	1.23 ± 0.09 b

^1^Half strength

^2^ Three quarters strength

^3^ Full strength. ANOVA and HSD Tukey test post-hoc analysis. Different letter in the same line indicates statistically significant differences between treatments (mean ± SD, N  =  10, Significance level was fixed at p<0.01.

### Induction of hairy roots and genetic transformation efficiency

**Fig 1:**
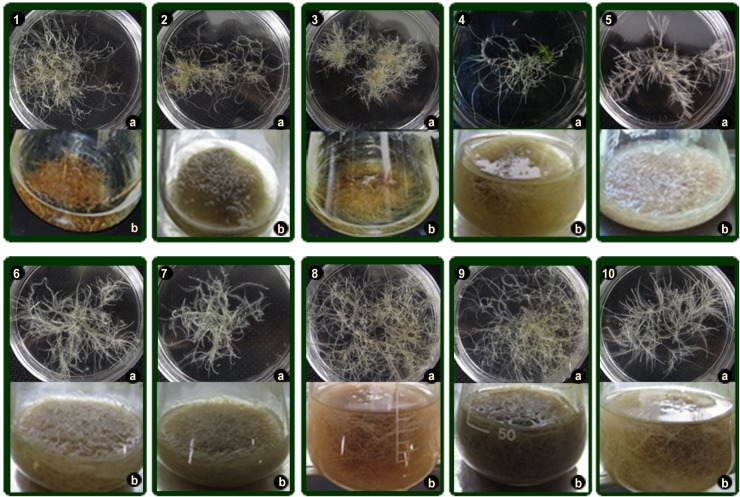
Ten selected HR lines. Individualized in solid MSB5 culture medium (1a – 10a) and in liquid MSB5 liquid culture medium (1b – 10b) without hormones.

In response to *Agrobacterium* infection (strains K599+pGus-GFP+ and ATCC15834+pTDT), putative hairy roots sprouted after 8–12 days in culture. In total, 144 putative hairy roots were induced; 15 days after infection, the roots were excised and placed in Petri dishes (five to six per Petri dish) containing hormone-free MSB5 culture medium (M0404 Sigma). Ten induced roots that grew faster were individually transferred to Petri dishes. The roots showed the characteristic hairy root phenotype of the *Agrobacterium rhizogenes*-transformed roots and high branched, rapid and plagiotropic growth, which was observed in both semisolid (Fig 1 HR-lines 1a – 10a) and liquid culture medium (Fig 1 HR-lines 1b – 10b).

The efficiency of transformation obtained with the ATCC15834+pTDT strain was 60% and with the K599+pGus-GFP+ strain was 46.7% (Table 2). One hundred forty-four putatively transformed roots were obtained, and ten HR lines with optimum growth were selected and further subjected to cytotoxic evaluation and PTOX identification by HPTLC.

**Table 2:** Number of induced and selected HR-lines.

Strain	Number of explants	Explants with roots sprout	Number of roots induced	Number of selected lines
K599+GFP-GUS+	15	7	34	4
ATCC15834+pTDT	40	24	110	6

**Fig 2:**
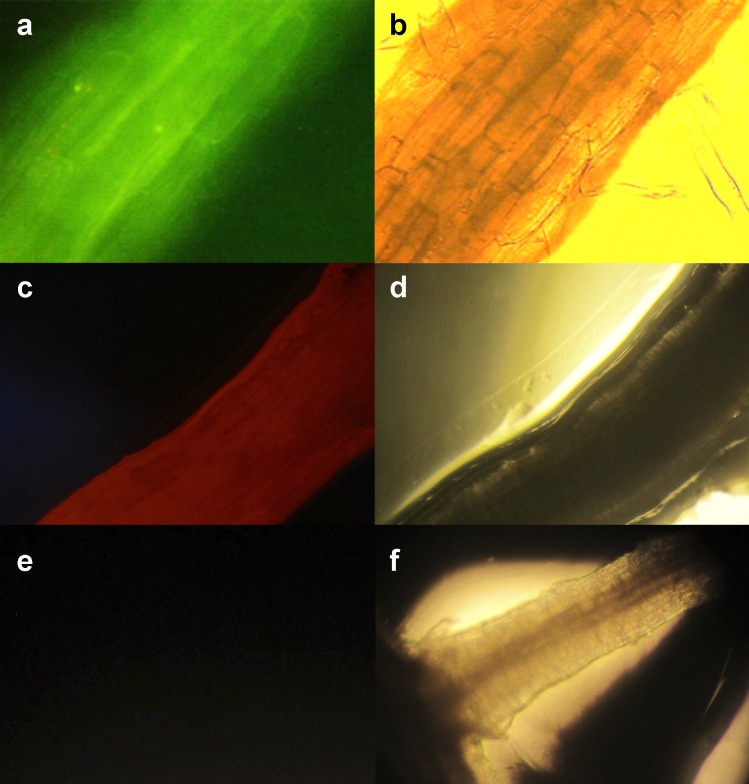
Confirmation of genetic transformation. Genetic transformation was corroborated by epifluorescence of induced hairy roots of *Hyptis suaveolens* with the strain K599+pGus-GFP+ (**a**, and **b**) and the strain ATCC15834+pTDT (**c**, and **d**). Observed in darkness and illuminated with blue (**a**), green (**c**), and white lights (**b**, **d**, and **f**). When nontransformed roots were illuminated with blue or green light, epifluorescence was not observed (**e**). All microphotographs are 20X.

Green or red epifluorescence was utilized to corroborate the genetic transformation of hairy roots. Four HR-lines expressed the green fluorescent protein (HsGF1 –HsGF4), and six expressed the threonine deaminase of tomato protein (HsTD5 –HsTD10), which fluoresces in red (Fig 2).

### Extraction and identification of PTOX utilizing HPTLC-Scan

**Fig 3:**
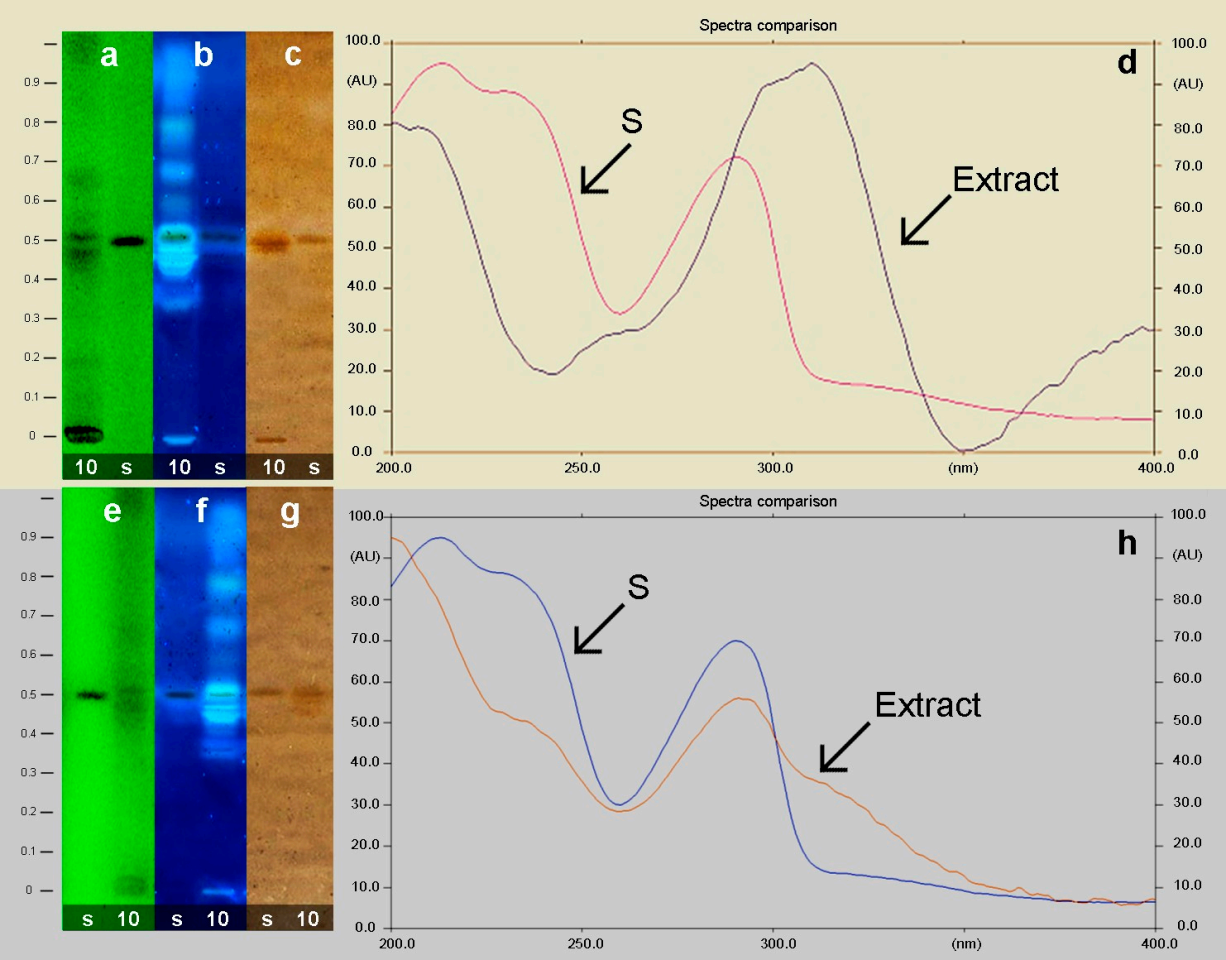
Identification of PTOX by HPTLC-Scan. Lane 10 indicates the HsTD10 HR-line extract, and the standard (POTX, Sigma) is referred to as letter “s”. The profiling of the different solvent extractions MeOH-CH_2_Cl_2_ (**a**, **b**, and **c**) and CHCl_3_ (**e**, **f**, and **g**) are presented. Developed-plate visualizations at 254 nm (**a,** and **e**), cerium sulfate revealed-plate at 366 nm (**b,** and **f**), and white light (**c,** and **g**) are shown. Absorbance spectra from the spots of PTOX in both extracts (**d,** and **h**) are shown.

The first extraction to identify PTOX included the use of both chloroformic and MD methods [19]. For the identification of PTOX by high performance thin layer chromatography (HPTLC) [20] of the ten HR lines, we considered the retention factor (Rf = 0.56) developed by the PTOX standard (Sigma). The displayed Rf = 0.53 of HsTD10 HR-line extracts was similar to the standard (Fig 3). The HPTLC profiling of HsTD10 HR-line extract visualized at 254 nm (Fig 3A and 3E) for the plate cerium sulfate-revealed and observed at 366 nm (Fig 3B and 3F), and we observed with white light (Fig 3C and 3G), indicated a very close RF with the PTOX standard (lane “s”). The identity of PTOX in the extracts was confirmed by the UV spectra profiling, generated by the Scanner–TLC 3-CAMAG) observed in Fig 3D (MeOH-CH_2_Cl_2_-extraction) and 3h (CHCl_3_- extraction), displaying a clear result in the last one.

### Cytotoxic evaluation

Both extracts from the ten HR lines were evaluated for cytotoxicity using the sulforhodamine (SRB) method. Cytotoxic evaluation showed that all of the extracts were cytotoxic for at least one carcinoma cell line. Depending on the extract, the HR-lines showed differential cytotoxic activity (Table 3). Some IC_50_ values were lower than 4 μg mL^-1^, a threshold value reported for active pure compounds [22]. The chloroformic and methanol-dichloromethane extracts from the HsTD10 HR-line showed the highest cytotoxic activity in three (SIHa, PC3, and MCF7) out of four carcinoma cell lines evaluated, with IC_50_ values ranging between 1.8 and 16.6 μg mL^-1^; therefore, this line was selected for continued research exploring and improving PTOX accumulation.

**Table 3:** Cytotoxicity (IC_50_ μg mL^-1^) of ten selected HR-lines of *Hyptis suaveolens*.

HR-Lines	Methanol–Dichloromethane extracts				Chloroformic extracts			
	Carcinoma cell lines* (IC50 μg mL^-1^)							
	HF6	SIHa	PC3	MCF7	HF6	SIHa	PC3	MCF7
HsGF1	5.9 ± 0.3	>20	>20	4.2 ± 0.1	6.4 ± 0.5	17.4 ± 0.9	>20	5.0 ± 0.3
HsGF2	6.7 ± 0.1	>20	>20	4.1 ± 0.4	3.2 ± 0.2	>20	>20	5.2 ± 0.4
HsGF3	7.1 ± 0.3	>20	>20	4.6 ± 0.3	12.1 ± 0.3	6.6 ± 0.7	>20	6.2 ± 0.5
HsGF4	11.7 ± 0.2	>20	>20	3.8 ± 0.2	10.9 ± 0.8	6.1 ± 0.2	>20	4.0 ± 0.3
HsTD5	5.5 ± 0.5	>20	>20	8.9 ± 0.6	15.7 ± 0.7	6.3 ± 0.1	>20	7.4 ± 0.6
HsTD6	7.7 ± 0.6	12.2 ± 0.5	>20	6.3 ± 0.8	14.2 ± 0.9	16.4 ± 0.2	>20	7.0 ± 0.8
HsTD7	2.8 ± 0.1	13.5 ± 0.3	>20	9.6 ± 0.5	5.1 ± 0.2	9.0 ± 0.4	18.5 ± 0.8	3.5 ± 0.4
HsTD8	18.1 ± 0.5	>20	>20	>20	>20	9.69 ± 0.6	>20	5.5 ± 0.3
HsTD9	9.1 ± 0.2	16.8 ± 0.7	>20	4.9 ± 0.2	9.1 ± 0.7	4.1 ± 0.2	16.2 ± 0.7	2.9 ± 0.7
**HsTD10**	**5.9 ± 0.2**	**7.1 ± 0.4**	**16.6 ± 0.7**	**3.3 ± 0.2**	**8.2 ± 0.4**	**1.8 ± 0.1**	**6.68 ± 0.3**	**2.5 ± 0.2**
PTOX	0.011	1.66E-03	1.2 ± 0.01	4.23E-6	0.011	1.66E-03	1.2 ± 0.01	4.23E-6
Etoposide	1.8 ± 0.1	0.35	0.99	0.03	1.8 ± 0.1	0.35	0.99	0.03

* Carcinoma cell lines: HF6 = Colon, SiHa = Uterine, PC3 = Prostate, MCF7 = Breast. The positive controls utilized were PTOX and Etoposide.

### Identification of PTOX using HPLC-MS and MicrOTOF-Q II in the HsTD10 HR-line

#### HPLC-MS

**Fig 4:**
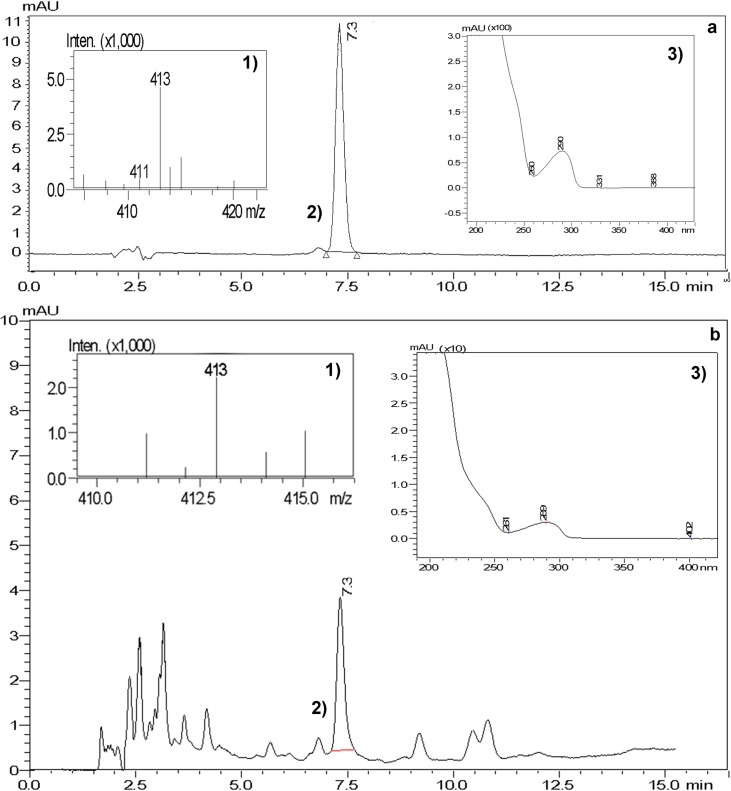
Identification of PTOX by HPLC-MS. (**a**) PTOX standard (Sigma) showing 1) molecular ion m/z 413 in negative mode, 2) retention time of the peak (7.3 min), and 3) UV spectrum absorption; (**b**) HsTD10 HR-line 1) molecular ion m/z 413 corresponding to PTOX in negative mode, 2) retention time of the peak at 7.3 min, and 3) UV spectrum similar to PTOX.

The identification of PTOX in the HsTD10 HR-line was also carried out by HPLC-MS. Fig 4 shows the chromatogram of the m/z 413 that corresponds to PTOX in a negative mode.

#### MicrOTOF-Q II

**Fig 5:**
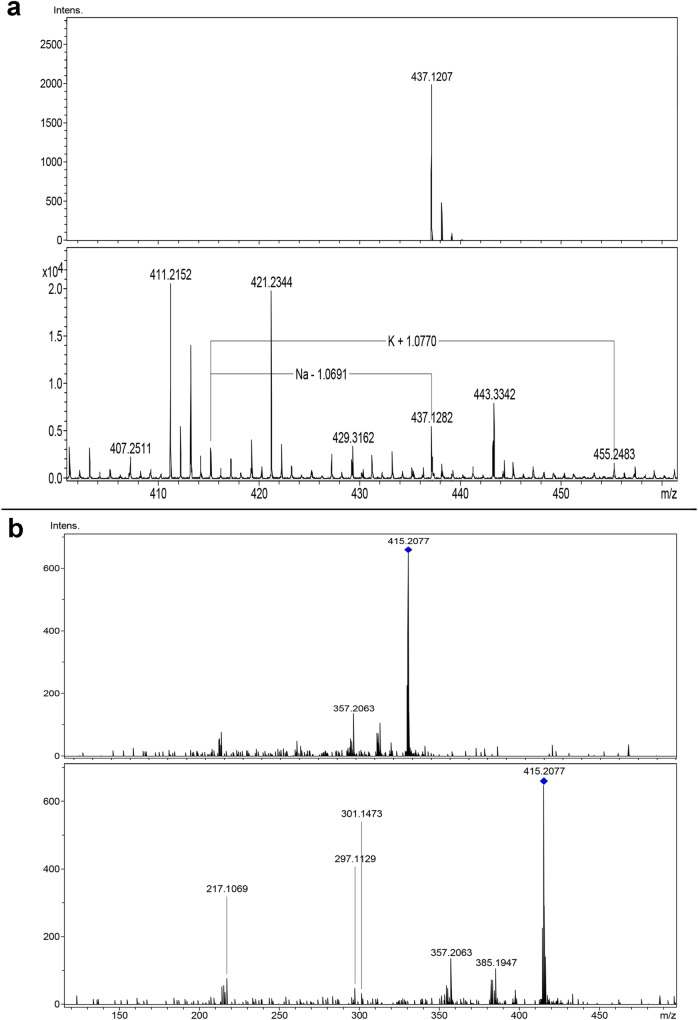
Identification of podophyllotoxin by micrOTOF-Q II in the HsTD10 HR-line. (**a**) spectrum of the extract showing the PTOX-Na and PTOX-K adducts, (**b**) partitioning profile (MS/MS) from molecular ion m/z 415 corresponding to PTOX (positive mode).

When the identification was performed by micrOTOF-Q II, there was overwhelming evidence that the selected HR-line produces PTOX, as the molecular ion m/z 415 corresponding to PTOX (positive mode), the molecular ion m/z 437 corresponding to the adduct PTOX-Na and the partitioning profile of m/z 415, were observed (Fig 5).

#### Competitive fragmentation modeling for metabolite identification (CFM-ID) platform

**Fig 6:**
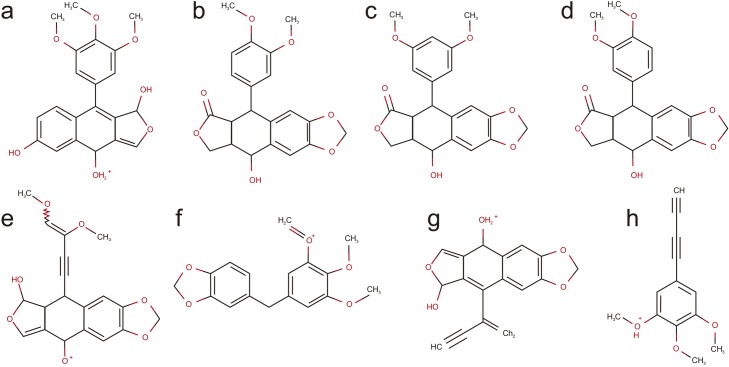
Structures predicted by CFM-ID related to each peak of partitioning profile from m/z 415 ion. (**a-d**) structures for m/z 385, (**e**) structure for m/z 357, (**f**) structure for m/z 301, (**g**) structure for m/z 297, (**h**) structure for m/z 217.

To correlate the observed peaks in the partitioning profile obtained by MS/MS from m/z 415 with predictive structures, the CFM-ID (http://cfmid3.wishartlab.com) platform was utilized, showing resulting ionized structures (Fig 6).

### Modification of culture conditions and yield of PTOX in the HsTD10 HR line

#### Effect of culture medium and of the method of extraction on yield of PTOX

The results showing PTOX accumulation in the HsTD10 HR line at 30 days of culture grown in MS, MSB5, and B5, all at TQS, are presented in Table 4. When the extraction of PTOX was performed using chloroform, a higher accumulation of this lignan was obtained from roots grown on MS medium followed by those grown on MSB5 and B5 media. However, when the extraction was undertaken using the modified MD method, the yield of PTOX increased considerably. Nevertheless, in this experiment, the best result was recorded in MSB5 followed by B5 and MS (Table 4).

**Table 4:** Influence of culture medium and extraction method in PTOX yield by the HsTD10 HR-line.

Culture medium at TQS	Concentration of PTOX (mg g^-1^ DW)		
	Extraction method		
	Chloroform	Koulman	Koulman Modified
MS	1.52 ± 0.011 a	1.99 ± 0.021 b	2.89 ± 0.035 b
MSB5	0.63 ± 0.017 b	**2.97 ± 0.027 a**	**4.50 ± 0.032** a
B5	0.58 ± 0.006 b	2.20 ± 0.014 b	3.24 ± 0.032 b

ANOVA and HSD Tukey test post-hoc analysis. Different letter in the same column indicates statistically significant differences between treatments (mean ± SD, N  =  3, Significance level was fixed at p<0.01).

#### Effect of thiamine addition to TQS MS culture medium on modulating the yield of PTOX in the HsTD10 HR-line

**Fig 7:**
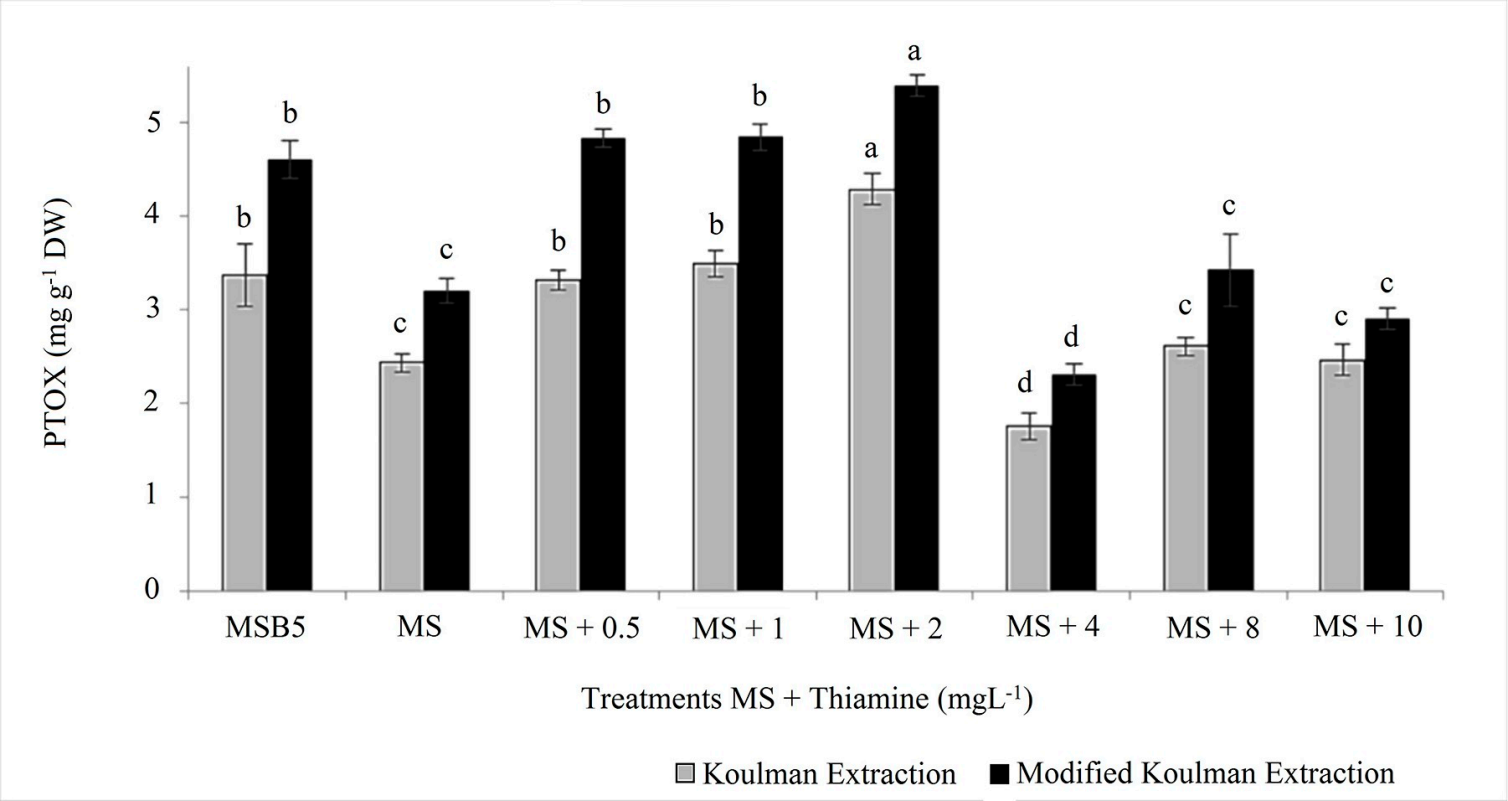
Effect of thiamine extra addition to TQS MS and of the extraction method in the production of PTOX by the HsTD10 HR-line at 30 days in culture. Koulman method (Gray) and modified Koulman method (Black) ANOVA, and HSD Tukey test post hoc analysis. Different letters indicate statistically significant differences between treatments (mean ± SD, N  =  3, Significance level was fixed at p<0.01).

The results regarding the effect on PTOX yield of extra addition of thiamine at various concentrations to the MS culture medium (0.5, 1, 2, 4, 8 and 10 mg L^-1^) are shown (Fig 7). When extraction was performed with sonication at 40 ± 5°C (Koulman modified), PTOX accumulation increased by 34% (Fig 7 black columns).

### Growth kinetics and PTOX accumulation

**Fig 8:**
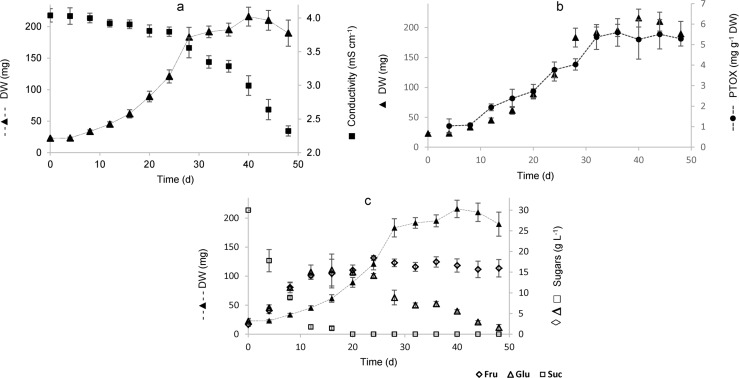
Kinetic parameters from the HsTD10 HR line during 48 days of growth in TQS MS medium supplemented with thiamine (2 mg L^-1^). (**a**) Correlation between dry weight (DW) and conductivity, (**b**) relation between biomass and PTOX accumulation, and (**c**) relation between carbohydrate consumption and biomass. (■ conductivity, —▲— DW, —●— PTOX, ◊ fructose, D glucose, □ sucrose).

Measurements of conductivity during growth kinetics showed that as anticipated, this parameter declined in inverse proportion to biomass increase. The highest dry weight biomass was achieved at day 40 (7.2 g L^-1^ DW), and the exponential phase of growth of the selected HR-line occurred from day 8 to day 28 with a mean specific growth rate (μ) of 0.11 d^-1^ (Fig 8A). The production of PTOX was growth associated, observing a maximum accumulation of 5.4 and 5.6 mg g^-1^ between days 32 and 36 (Fig 8B). Concerning carbohydrate consumption, sucrose concentration on the medium decreased at a constant rate until day 20, when it was completely hydrolyzed; in contrast, fructose and glucose initially increased up to day 20, and the concentration of glucose began to diminish thereafter, while fructose concentration remained unchanged (Fig 8C).

## Discussion

### Effect of medium components on plantlet regeneration

The best result for regenerated plantlets evaluated by height and root length was obtained with TQS MS medium. Regenerated plantlet height was similar for 15 days, but subsequently, significant differences (p<0.01) between all treatments emerged. In contrast, roots were sprouting on TQS MS at day 10, although when half-strength (HS) or full-strength (FS) MS were used, root sprouting occurred at 25 and 30 days, respectively. Likewise, sprouted roots in TQS MS at 30 days grew up to five to fifteen times the length compared to those grown at FS MS and HS MS, respectively. This result is similar to that reported in 2011 by Borges et al. [24] for the root length and plantlet height of *Discorea alata* but different from that reported in 2002 by Espinosa et al. [25] and in 2016 by Jiménez-Mariña et al. [26], who obtained better results with FS MS in terms of plantlet height for *Ipomea batatas* and *Dianthus caryophyllus*, respectively. There is a direct relationship between plantlet height and root length.

### Genetic transformation efficiency and its corroboration

Transformation efficiency (defined as the number of responsive explants regarding the total number of infected explants) obtained in the present work using ATCC15834+pTDT (60%) and K599+pGus-GFP+ (46.7%) strains was greater than that obtained in 2013 by Ooi et al. [27] with the A4 *Agrobacterium* strain infecting *Solanum mammosum* (21.4%) but lower than that reported in 2015 by Thilip et al. [28], with the R1000 *Agrobacterium* strain infecting *Withania somnifera* (93.3%).

The corroboration of transformation has traditionally been reported by Southern blotting or using PCR. However, in this work, the use of K599+pGus-GFP+ and ATCC15834+pTDT strains enabled confirmation of transformation by visual observation of fluorescence through an epifluorescence microscope. The most important advantage of this method is that it reduces the carbon footprint and does not damage the obtained transformed tissues. Moreover, this approach has been successfully utilized for the structural elucidation of biological molecules and their interactions, as well as for *in vitro* assays or *in vivo* monitoring cellular research [29].

### Extraction and identification of PTOX utilizing HPTLC-Scan

The identification of PTOX in wild roots of *H*. *suaveolens* has been reported using high-performance liquid chromatography coelution [16] and liquid chromatography-masses (m/z 415, positive mode) [15]. Similarly, the adduct PTOX-Na (m/z 437) has also been reported in leaves of *Juniperus bermudiana* [5]. In the present work, we used the methodology described by Kamal et al. [20], who reported the identification and quantification of PTOX using an HPTLC system. It was possible to confirm the presence of PTOX in the extracts by the development of spots with similar Rfs: 0.56 for the PTOX standard and 0.53 for the extracts. The slight difference in Rfs can be explained because the extract is a complex matrix composed of many metabolites which, in turn, influence chromatographic development. Nevertheless, the unequivocal identity of PTOX in the extracts was also confirmed through spectroscopic (maximum UV absorption) and spectrometric (LC-MS/MS) evidence.

### Cytotoxic evaluation

All HR-lines were cytotoxic for at least one of the carcinoma cell lines utilized. Interestingly, IC_50_ values were lower than 4 μg mL^-1^ in five out of ten evaluated HR lines, which is propitious, as this value is a predicted value for a pure compound [22]. The HsTD10 HR-line was the most cytotoxic in three (SIHa, PC3, and MCF7) out of four carcinoma cell lines, and the one that showed activity against all the evaluated carcinoma cell lines.

### Identification of PTOX using HPLC-MS and MicrOTOF-Q II in the HsTD10 HR-line

To corroborate the identification and quantification of PTOX in the extracts of the HsTD10 HR-line, we used HPLC-MS equipment in negative mode (m/z 413) because under our ionization conditions, the m/z 415 (positive mode) appeared to be notably low. The m/z 415 molecular ion was identified with a micrOTOF-Q II mass spectrometer.

This equipment permitted the identification of the adduct PTOX-Na (m/z 437), as reported in 2011 by Renouard et al. [5], and the partitioning profile during the MS/MS experiment of the m/z 415. This result is similar to several spectra available at the https://pubchem.ncbi.nlm.nih.gov/compound/podophyllotoxin#section=MS-MS site [23]. Seven of the eight predictive structures proposed as resulting from ionization of the PTOX molecule in this work can also be observed, following links (4.3.2. MS/MS spectrum 108322, 108323, 175510, and 175511). In both cases, prediction was carried out utilizing the Competitive Fragmentation Modeling for Metabolite Identification platform (CFM-ID) from Wishart-lab (http://cfmid3.wishartlab.com).

### Selection of culture conditions and yield of PTOX in the HsTD10 HR-line

Under our conditions, the culture medium affected the concentration of PTOX, which was determined on the HsTD10 HR-line growing on three different media. It has been reported that the best culture medium for improving PTOX production depends on the plant species and the type of *in vitro* cultivation employed. The influence of MS and B5 (Gamborg’s) culture media for biomass growth and PTOX accumulation on transformed cell suspension and hairy root cultures of *Linum album* [11], as well as in hairy root cultures of *L*. *flavum*, higher biomass accumulation and PTOX production were accomplished with B5 medium [13]. In contrast, MS medium has been utilized for the production of PTOX and MPTOX in adventitious root cultures of *Podophyllum peltatum* [9], hairy root cultures of *Linum mucronatum* ssp. *mucronatum* [30], and hairy root cultures of *Linum strictum* ssp. *strictum* [31].

#### Effect of culture medium and the extra addition of thiamine on PTOX accumulation

Considering the results described in this study, it was necessary to ascertain how much the culture medium or the method of extraction affected the yield of PTOX in the HsTD10 HR-line. When the HsTD10 HR-line was established in three culture media and extraction was performed using chloroform, the best result was obtained in roots grown in MS. However, when the extraction was performed using the modified MD method, the best result was obtained from roots grown in MSB5 medium (7 times more PTOX than that obtained in chloroform extracts from the same line, growing in the same culture medium). The differences between MS and MSB5 culture medium pertain to the concentration of vitamins: 100 times more thiamine and twice the amount of pyridoxine and nicotinic acid in the MSB5 medium. Then, the question was whether thiamine could modulate PTOX production. The addition of thiamine to TQS MS medium increased the yield of PTOX in the HR-line.

Our results showed that there was no significant difference in biomass production, but there was a clear difference in PTOX accumulation. The increase in PTOX production was dose dependent on the thiamine concentration up to a maximum of 2 mg L^-1^; at this point, production began to decrease with each succeeding increased dose of thiamine.

To date, the relationship between the addition of thiamine to the culture medium and the increase in PTOX production has not been reported. Lignans are phenylpropanoid compounds that have been identified as plant-defenders [32–34]. Regarding the phenylpropanoid pathway, Boubakri et al. [35] reported that thiamine increased the expression of genes along this route in grape vines. This vitamin increased plant defense activity [36], induced systemic acquired resistance (SAR) in *Arabidopsis*, rice, tobacco, and cucumber [37], and increased the concentration of callose and lignin in *Arabidopsis* [38].

As the accumulation of PTOX in the HsTD10 HR-line was dose-dependent up to 2 mg L^-1^ of thiamine, and higher concentrations notably reduced the accumulation of PTOX, in future experiments, it would be interesting to evaluate enzyme activity in the cultures of this HR-line. The increase of thiamine in the HsTD10 HR-line of *Hyptis suaveolens* may activate a defense mechanism, possibly increasing the concentration of some phenylpropanoids but definitely that of PTOX.

#### Effect of sonication at 40 ± 5°C in PTOX extraction

Several reports have analyzed the effect of temperature on PTOX extraction. In 2002, Bedir et al. [6] reported greater PTOX extraction from leaves of *Podophyllum peltatum* and modified leaves and twigs of *Juniperus virginiana* using hot methanol. However, in 2011, Renouard et al. [5] analyzed the effect of temperature but did not report any significant differences in *Juniperus bermudiana* or in 12 other species from the same genus; nevertheless, in these reports, sonication was not employed. It is important to note that the modified MD method was performed as described in 2003 by Koulman et al. [19] with one change, as instead of performing sonication for one hour at room temperature, we sonicated for one hour at 40 ± 5°C. This change significantly increased PTOX extraction. In our work, it is clear that the culture medium and the extraction method were factors that determined the level of PTOX production.

### Growth kinetics

The highest dry weight biomass obtained under our culture conditions at day 40 was 9.4 times higher than the inoculum. The specific growth rate of the HsTD10 HR-line suspension cultures (0.11 d^-1^) is higher than that reported in 2006 by Nader et al. (0.08 d^-1^) [39] but lower than that reported in 2005 by Caspeta et al. (0.12 d^-1^) [40] for hairy root cultures of *Galphimia glauca* and *Solanum chrysotrichum*, respectively. Moreover, the production of PTOX associated with growth observed in this work is similar to that observed in hairy roots and in cell suspension cultures of *L*. *album* [11, 41], as well as in cell suspension cultures of *P*. *hexandrum* [42].

In this work, the consumption of carbohydrates was selective for sucrose and glucose because fructose was not consumed. This result is similar to that reported in 1998 by Hammouri et al. [43], who found that the hairy roots of *Beta vulgaris* were unable to use fructose as a source of energy when using the same initial concentration of sucrose (3%). Nonetheless, this result differs from that reported in 2000 by Shimon-Kerner et al. [44] because they found similar levels of glucose and fructose in hairy root cultures of *Symphytum officinale* after 28 days in culture, a difference which is probably due to the time period of their kinetic experiment.

It is also important to note that the concentration of PTOX between days 32 and 36 at the beginning of the stationary growth phase was the highest obtained (5.4 and 5.6 mg g^-1^, respectively).

PTOX accumulation in wild plants and plant material obtained by *in vitro* cultures (cell, callus, tissues, plantlets, transformed tissues) show a different pattern. To date, the greatest supply of PTOX has been obtained from the wild plant *Podophyllum hexandrum* (43 mg g^-1^ DW) [1]; however, this yield diminishes when PTOX is obtained from cell suspension cultures of this species (7.9 mg g^-1^ DW) [8]. Similarly, the concentration of PTOX in wild *Podophyllum peltatum* plants is similar to that obtained in cell suspension cultures (4.7 and 5.9 mg g^-1^ DW, respectively) [6, 9]. The accumulation of PTOX in *Linum album* has been reported to be higher in cell suspension cultures (8 mg g^-1^ DW) [7] than in wild roots of the same species (0.3 mg g^-1^ DW) [45]. However, the highest accumulation of PTOX in this species was obtained from hairy root extracts (15 mg g^-1^ DW) [10].

The highest concentration of PTOX reported using *in vitro* cultures was obtained in hairy root cultures of *Linum album* (15 and 5.1 mg g^-1^ DW) [10, 11] and *L*. *flavum* (4.5 mg g^-1^ DW) [13]. Under our conditions, the optimum yield obtained for the HsTD10 HR-line (5.6 mg g^-1^ DW) is similar to that reported in 2008 by Baldi et al. [11] in *L*. *album*, which is 100 times higher than that obtained from roots of wild plants and 56 times higher than that obtained from nontransformed roots of *Hyptis suaveolens* grown *in vitro* [16].

## Conclusions

Nodal explants of *Hyptis suaveolens* infected with K599+pGus-GFP+ and ATCC15834+pTDT strains from *Agrobacterium rhizogenes* were suitable explants for obtaining ten hairy root line cultures. The HsTD10 HR-line was the greatest cytotoxic and podophyllotoxin producer. The culture medium did not significantly affect biomass growth but was a determining factor for the accumulation of PTOX in the hairy roots of *H*. *suaveolens*, an influence that was greatest when using MS at three-quarters strength. Thiamine modulated the production of PTOX, obtaining the highest values when it was added at 2 L^-1^ to MS medium. Sonication at 40 ± 5°C using MD extraction increased PTOX extraction. PTOX accumulation in kinetic suspension culture of *H*. *suaveolens*-hairy roots was growth-associated. The accumulation of PTOX in the HsTD10 HR-line was greater than that obtained from the roots of wild plants or *in vitro* nontransformed cultured roots.
